# Implementation of Teledermatology in Alberta, Canada: A Report of One Thousand Cases

**DOI:** 10.1177/12034754221108990

**Published:** 2022-07-08

**Authors:** Cristina Olteanu, Melika Motamedi, Jessica Hersthammer, Brandon Azer, Jaggi Rao

**Affiliations:** 1 3158 Division of Dermatology, University of Alberta, Edmonton, Canada; 2 Faculty of Medicine and Dentistry, University of Alberta, Edmonton, Canada; 3 Faculty of Sciences, University of Alberta, Edmonton, Canada

**Keywords:** teledermatology, telemedicine, teleconsulting, consultDerm

## Abstract

**Background:**

Teledermatology utilizes photoimaging and background information to allow dermatologists to remotely provide a diagnosis to practitioners. ConsultDerm is an asynchronous, web-based teledermatology software that allows practitioners to submit their electronic referrals for assessment by board-certified dermatologists.

**Objective:**

Our study aimed to retrospectively analyze teledermatology’s utilization in Canada by using the teledermatology platform ConsultDerm.

**Methods:**

After implementing inclusion criteria, 1000 patients were selected, and relevant demographic and clinical information were extracted for data analysis. In addition, an online survey with pre-formulated questions was distributed to 7 dermatologists currently using the ConsultDerm platform to determine their experience in utilizing teledermatology.

**Results:**

Of the 1000 patients, 66.5% had not received treatment prior to their teledermatology referral, and on average, patients experienced symptoms for 489.5 days prior to their referral. Diagnoses made were categorized by conditions, most common being dermatitis (37.1%), followed by acneiform conditions (10.6%), benign lesions/neoplasms (12.1%), infections (9.4%), and dyspigmentation (3.1%). Most consults originated from small population centers, and the referring practitioners were predominantly family physicians. Dermatologists utilizing the platform expressed ease of use, however, areas of improvement were identified such as increasing the quality of imaging and more detailed patient history.

**Conclusion:**

Through our analysis of 1000 cases, we identified how a teledermatology consultation could be used to assess a wide variety of cutaneous conditions, improving access for patients who may face barriers to seeing a dermatologist.

## Introduction

With the ever-evolving landscape of technology, the utilization of telemedicine is an emerging field in healthcare that has a tremendous level of potential to provide care to underserved areas and patient populations. Teledermatology, considered a subspecialty of dermatology, utilizes photoimaging and patient history to allow dermatologists to provide a diagnosis to practitioners or patients remotely. This form of telemedicine has had significant growth in its utilization over the past several years.^1^ This delivery method has provided a platform for physicians of various specialties, with an emphasis on general practitioners, to communicate with experts in dermatology in the care of their patients. The use of teledermatology has demonstrated efficiency in reducing wait times for patients, remedying less urgent referrals, and in light of the current COVID-19 pandemic allowing urgent reviews to occur and providing invaluable advice for high-risk patients, such as those on immunomodulators.^2,3^ A systematic review by Finnane et al. reported that teledermatology services consistently reduced wait times and improved satisfaction with care.^4^

In a 2019 report provided by the Canadian Medical Association (CMA), there were 634 dermatologists in Canada, of which 63 reside in Alberta, providing one dermatologist per 150,000 Albertans.^5^ As the number of experts in dermatology is usually scarce in comparison to their demand, with a striking disparity in dermatologist density between urban and rural areas, the implementation of teledermatology is especially helpful in providing care to remote areas. It is also instrumental to those who face physical barriers to care, such as limited transportation.^6^ The use of teledermatology for patients in remote areas has been proven beneficial.^6^ A systematic review by Lee and English found that in-person referrals were reduced by up to 74% when a photo-triage system was utilized.^7^ Also, a randomized study by Datta et al. found that teledermatology was significantly more affordable than conventional in-person physician-patient meetings.^8^ While teledermatology has the potential to provide aid in many different community-based settings, the implementation of a teledermatology triage system in a hospital-based setting was also proven to be successful, creating more avenues for the utilization of this technology.^9^

**Figure 1:**
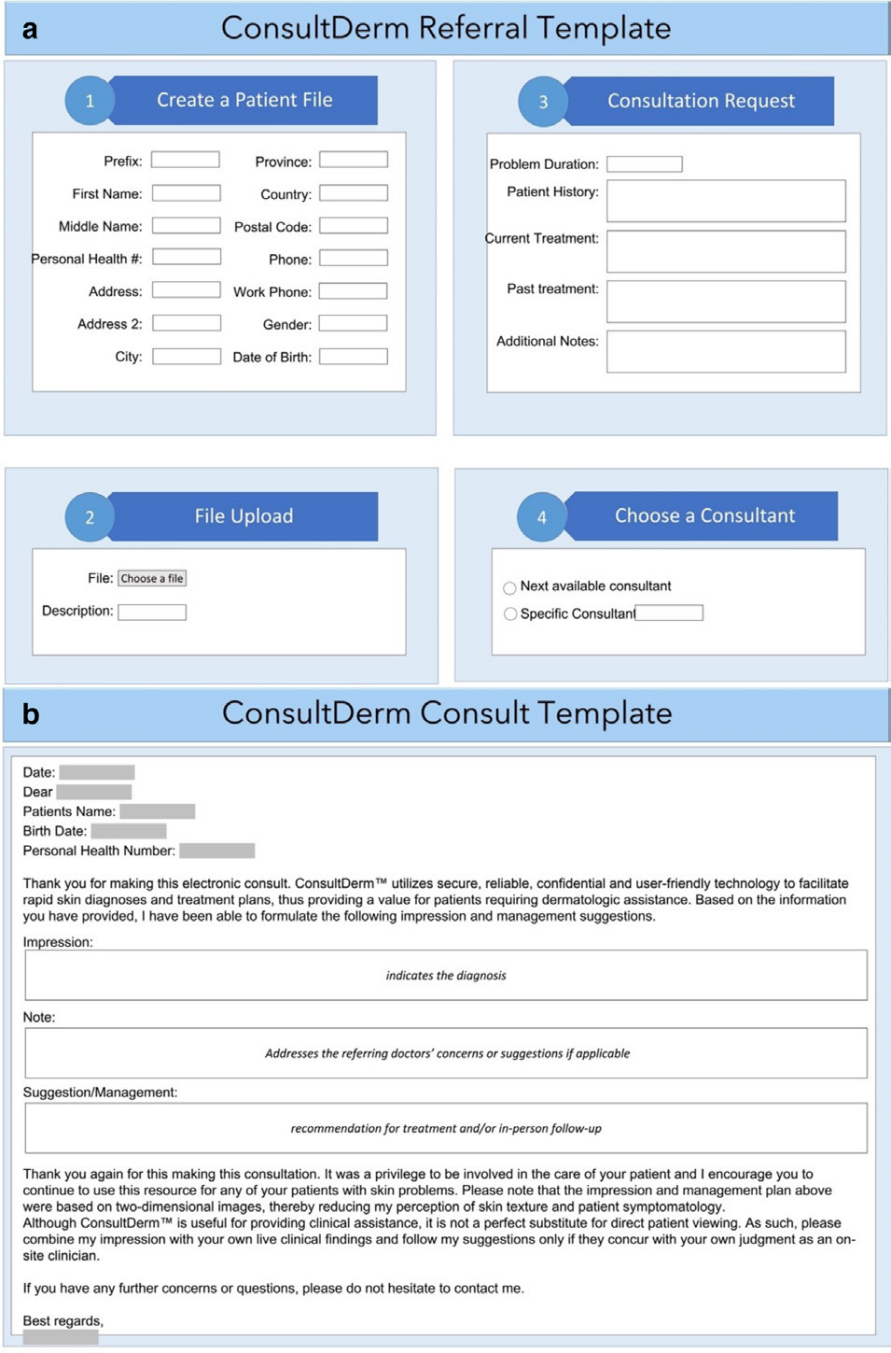
Template for referral (a) and consultant (b) on ConsultDerm. Boxed areas indicate customizable section of the template.

Technology has changed the way we deliver care in various ways. As a result of the current COVID-19 pandemic, telemedicine, including teledermatology, is a crucial tool in providing care to immunocompromised and at-risk patients. Through the asynchronous teledermatology platform in Canada, “ConsultDerm” (www.consultderm.com), patient images along with demographic information and pertinent history are stored and forwarded by the referring physician through the online platform to await a dermatologist consultant’s opinion. A referral and consultant template seen on ConsultDerm are provided in Figure 1. ConsultDerm is a web-based teledermatology platform, which allows practitioners, specifically physicians and nurse practitioners across Canada, to consult board-certified dermatologists regarding their patients at no cost to the referring practitioners. There are twelve teledermatologists on ConsultDerm providing consultation who are licensed and practicing dermatologists in Canada. These dermatologists supplement their in-person practice with teledermatology by way of ConsultDerm. Dermatologists using the ConsultDerm platform can seek remuneration through fee-for-service billing per consultation, which is paid to the practitioner by provincial healthcare in the patient’s home province. In the event of a consultation, the referring practitioner is given a template to provide the duration of the problem, a patient history, anatomic sites affected, and current and past treatments. There are also multiple spaces available for practitioners to upload images for the dermatologist to review. Responses by the dermatologist will include their diagnosis, an explanation of the disease, and a recommended treatment approach. It is at the discretion of the referring practitioner to initiate the recommended treatment. If they are uncomfortable doing so, they can then refer the patient. The consultation request is straightforward and has no specific requirements in terms of methodology for photo capture; however, if the dermatologist is unable to interpret the picture due to quality issues, they will request a new photo be uploaded in their response. In addition, there are no inclusion/exclusion criteria guiding a consult for referring practitioners. However, suppose a dermatologist believes that a teledermatology consult is insufficient to make a proper assessment or further investigations are required. In that case, they advise the referring practitioner to make an in-person referral for the patient to a dermatologist and provide their office location if they choose to refer there. As patient information is collected on the platform, consent templates are available to referring practitioners to provide to their patients. Consultderm has been approved by the privacy commissioner and OIPC in Alberta and adheres to the health information act set forth in Canada.

To our knowledge, there are a limited number of studies assessing a large cohort of real-life cases that have been seen through practitioner-to-physician referral by the method of teledermatology and how these may differ from those seen in conventional office visits. Several teledermatology platforms are available in Canada, including Dermcafe, Maple, and DermaGo. The majority of these platforms have come to rise in the last ten years and operate on a patient-to-physician basis, meaning patients would describe their symptoms and submit photographs, later being contacted by a physician. However, the platform ConsultDerm has been around the longest of any web-based platforms, being operational since 2008, and operates as a practitioner-to-physician platform requiring background history and images from a referring provider. Consultderm is advertised to referring practitioners by means of their governing body and associations. Consultderm helps expedite referrals for community practitioners and has demonstrated applicability in acute settings, such as those in hospitals for an emergency dermatology consultation. In this paper, we discuss how teledermatology is being utilized by way of practitioner-to-physician e-referral in Canada by assessing its use through ConsultDerm in the province of Alberta. In this paper, we discuss how teledermatology is being utilized by way of practitioner-to-physician e-referral in Canada by assessing its use through ConsultDerm in the province of Alberta.

## Materials and Methods

Between May 25, 2020 and October 20, 2020, 1077 consults conducted in Alberta were extracted from the telemedicine platform ConsultDerm. Of those consults, 77 were excluded due to incomplete patient demographics and/or missing history of skin condition within the referral. One-thousand patient consults, satisfying all required information, were included, and used for data collection for this study.

Of the consults selected, information collected included age, sex, problem duration, any previous treatments were tried, the specialty of the referring physician, and how long it took for a diagnosis to be made from initial consult submission. Numerical measurements for quantitative data were calculated, including means, ranges, and percentages. Patient location was extracted and grouped into a population density category by identifying the location of that patient’s referring provider. These locations were categorized according to Statistics Canada as: (1) Rural (less than 1,000 population); (2) Small population centers (a population between 1,000 and 29,999); (3) Medium population centers (a population between 30,000 and 99,999); (4) Large population centers (a population of 100,000 or more).^10^

A 9-question voluntary survey using the online platform Survey Monkey was designed by one of the authors, a dermatology resident, and distributed to 7 dermatologist consultants that had previously used the ConsultDerm platform. The purpose of the survey was to inquire about both quantitative and qualitative elements, assessing the users’ experience, the platform’s practicality, and whether they noted any improvements which could be made. Of the dermatologists who participated in the survey, teledermatology is in addition to their regular in-office practices. As the dermatologist providers are not bound to provide care by the platform and may choose to stop at any time, the survey was sent to those providers who had provided care during the period the cases were completed. The survey questions are listed in Supplemental Table 1.

Ethics approval was obtained from the Health Research Ethics Board - Health Panel at the University of Alberta.

## Results

### Consults

Two hrundred and thirty-eight (23.8%) participants were pediatric patients (<18 years old) with a mean age of 7 years (range: 0-17). The other 762 participants (76.2%) consisted of adult patients, with a mean age of 51 years (range: 18-99 years). Five hundred and eighty-six (58.6%) patients were female, and 414 (41.4%) were male. Most of the patients (66.5%) had not received previous treatments prior to their ConsultDerm diagnosis. On average, patients experienced symptoms for 489.5 days (range: 1.5-10950) prior to their referral. Using ConsultDerm, the average time of diagnosis from initial referral was 5.2 days (range: 0-34). Of the 1000 consults, 5% were unable to be adequately assessed due to missing or poor image quality. For this in-person consultation was required for further assessment and investigation.

The diagnosis of the participants included in the study was categorized by condition (Table 1), the specifics of which can be found in Supplemental Table 2. The most common category of diagnosis was dermatitis (n = 371, 37.1%); followed by acneiform conditions (n = 106, 10.6%), benign lesions/neoplasms (n = 121, 12.1%), infections (fungal/yeast, bacterial, viral and ectoparasites, n = 94, 9.4%), and dyspigmentation (n = 31, 3.1%). All conditions have a higher proportion of adult patients to pediatric patients except for the fibromatoses category. However, it should be noted that skin cancer, bullous disease, and oral conditions were not seen in pediatric patients. The condition with the longest average problem duration prior to a ConsultDerm consult was psoriasis (2238.5 days, 6 years), and bullous diseases had the shortest average time (14.0 days). More patients had received treatment for their condition (62.4%) than not (37.6%) prior to ConsultDerm.

**Table 1:** Patient Demographics Categorized by Dermatological Conditions for 1000 patients From ConsultDerm.

Conditions	N	Average age in years (SE)	% (n)	% (n)	Sex % (n)		Previous treatments % (n)		Problem duration in mean days (SE)	Diagnosis time in mean days (SE)
			Peds (<18)	Adults (>18)	F	M	Yes	No		
Dermatitis	371	41.4 (1.3)	23.5% (87)	76.5% (284)	55.8% (207)	44.2% (164)	83.3% (309)	16.7% (62)	309.2 (37.0)	5 (0.3)
Acneiform conditions	106	31.1 (2.0)	29.3% (31)	70.8% (75)	62.3% (66)	37.7% (40)	78.3% 83)	21..7% (23)	654.1 (123.8)	4.3 (0.5)
Benign lesions/neoplasms	121	42.7 (2.8)	30.6% (37)	69.4% (84)	35.5% 43)	64.5% (78)	56.2% (68)	43.8% (53)	849.3 (166.0)	6.3 (0.7)
Infections	94	34.4 (2.4)	29.3% (28)	70.2% (66)	50.0% (47)	50.0% (47)	68.1% (1)	31.9% (30)	386.4 (72.6)	4.8 (0.5)
Fungal/yeast										
Viral										
Bacterial										
Ectoparasites										
Dyspigmentation	31	36.3 (3.1)	16.1% (5)	83.9% (26)	74.2% (23)	25.8% (8)	32.3% (10)	67.7% (21)	581.4 (169.8)	5.8 (1.4)
Vasculopathy/vasculitis/purpura	31	49.1 (4.3)	9.7% (3)	90.3% (28)	61.3% (19)	38.7% (12)	43.4% (15)	51.6% (16)	245.7 (101.7)	5.7 (1.0)
Other Papulosquamous	31	40.1 (4.4)	25.8%(8)	74.2%(23)	61.3%(19)	38.7%(12)	74.2%(23)	25.8%(8)	370.1 (176.3)	3.9 (0.7)
Psoriasis	30	44.2 (3.6)	6.7%(2)	93.3%(28)	36.7%(11)	63.3%(19)	93.3%(28)	6.7%(2)	2238.5 (580.2)	4.7 (0.9)
Other inflammatory skin diseases	50	41.1 (3.8)	24.0% (12)	76.0% (38)	64.0% (32)	36.0% (18)	70.0% (35)	30.0% (15)	121.6 (22.5)	4.8 (0.9)
Skin cancer	18	79.1 (3.2)	0.0% (0)	100.0% (18)	61.1% (11)	38.9% (7)	44.4% (8)	55.5% (10)	360.1 (84.3)	5.1 (1.8)
Nail disease (noninfectious)	19	38.5 (4.9)	21.1% (4)	78.9% (15)	57.9% (11)	42.1% (8)	36.8% (7)	63.2% (12)	560.1 (203.0)	3.2 (0.9)
Urticaria	15	32.3 (4.3)	13.3% (2)	86.7% (13)	80.0% (12)	20.0% (3)	80.0% (12)	20.0% (3)	306.4 (192.4)	4.7 (1.2)
Alopecia	11	31.6 (4.7)	27.6% (3)	72.7% (8)	81.8% (9)	18.2% (2)	18.2% (2)	81.8% (9)	165.5 (60.0)	4.9 (2.0)
Granulomatous	14	41.4 (7.2)	28.6% (4)	71.4% (10)	78.6% (11)	21.4% (3)	71.4% (10)	25.6% (4)	1450.3 (640.9)	9.7 (2.0)
External Trauma and Scars	9	49.7 (8.8)	11.1% (1)	88.9% (8)	77.8% (7)	22.2% (2)	11.1% (1)	88.9% (8)	983.8 (632.8)	11.6 (3.6)
Bullous diseases	7	85.7 (2.9)	0.0% (0)	100.0% (7)	71.4% (5)	28.6% (2)	100.0% (7)	0.0% (7)	14.1 (5.3)	8 (3.2)
Fibromatoses	5	25 (12.0)	60.0% (3)	40.0% (2)	40.0% (2)	60.0% (3)	40.0(2)	60.0 (3)	146.9 (100.1)	5.4 (3.2)
Oral conditions	2	56.5 (22.5)	0.0% (0)	100.0% (2)	0.0% (0)	100.0% (2)	100.0% (2)	0.0% (0)	298 (242)	0.5 (0.5)
Hypersensitivity reactions	27	36.5 (3.9)	18.5% (5)	81.5% (22)	66.7% (18)	33.3% (9)	66.7% (18)	33.3% (8)	55.7 (24.9)	4.9 (1.3)
Other	8	57.9 (7.9)	12.5% (1)	87.5% (7)	50.0% (4)	50.0% (4)	75.0% (6)	25.0% (2)	113.3 (61.9)	9.6 (2.2)

Abbreviations: F, female; M, male; N, number of patients; SE, Standard Error.

^a^Problem duration: The average number of days patients have been living with their condition at time of consult submission.

^b^Diagnosis time: The average number of days it took for a patient to receive their diagnosis from consult submission.

**Figure 2:**
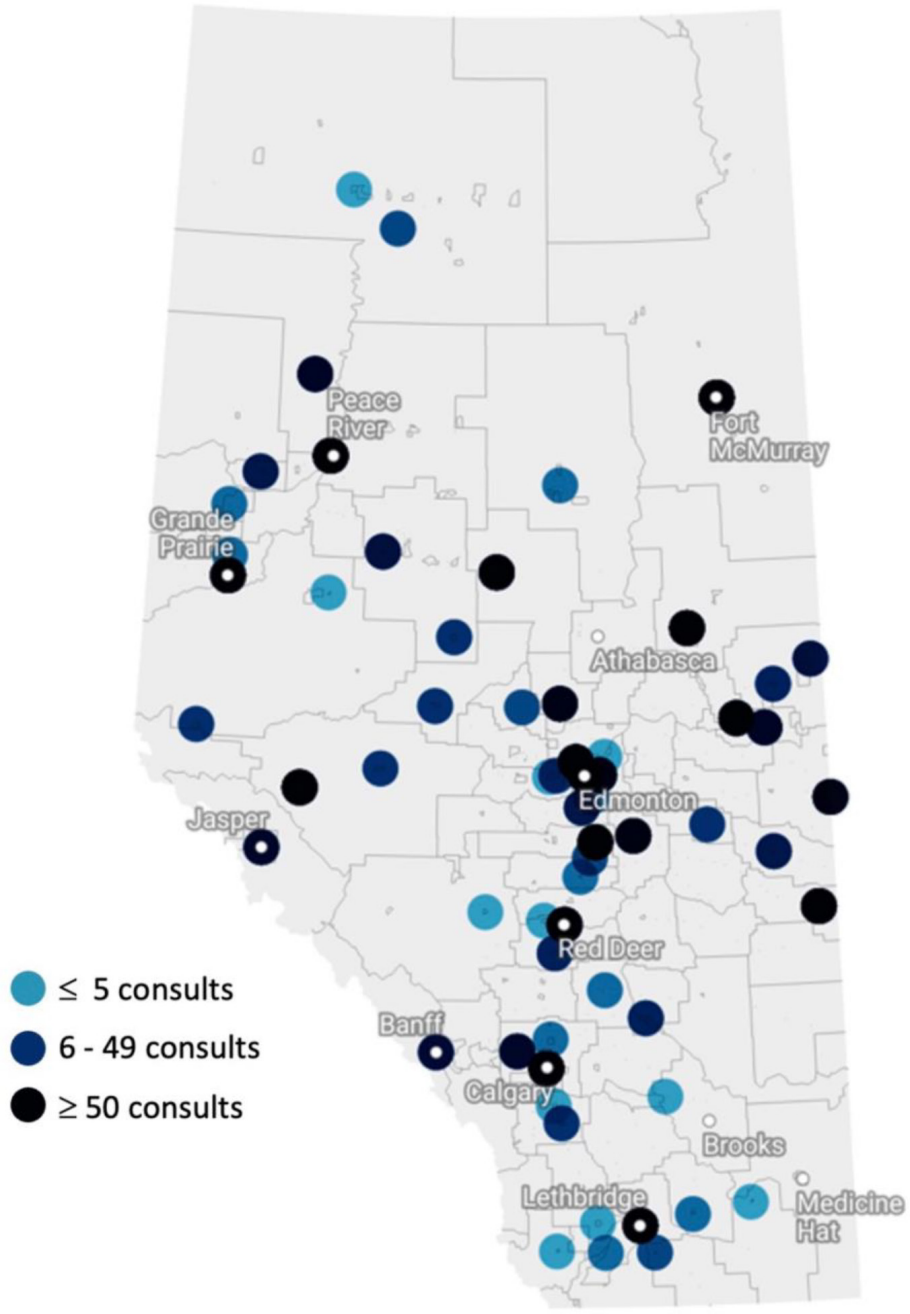
Map of Alberta displaying geographic locations of all 1000 consults.

As indicated in Table 2, the most significant proportion of referrals came from small population centers (45.4%), followed by large population centers (41.2%), medium population centers (12.6%), and lastly, rural areas (0.08%). Additionally, smaller population sizes correlated with fewer dermatologists within the region; rural areas had zero dermatologists, whereas 70 dermatologists resided in large population centers. A map of Alberta in Figure 2 illustrates where referrals originated from.

**Table 2:** Distribution of Location Type (Based on Population Size) Where First Consultation Occurred for ConsultDerm.

Population	Consult (n)	Percentage (%)	Dermatologist (n)
Rural Area^a^	8	0.08	0
Small POPCTR^b^	454	45.4	1
Medium POPCTR^c^	126	12.6	2
Large POPCTR^d^	412	41.2	70

Abbreviation: POPCTR, population centres.

The populations were categorized in the following way:

The number of dermatologists found in each population is indicated.

^a^Rural area (less than 1,000 population),

^b^Small POPCTR (a population between 1,000 and 29,999)

^c^Medium POPCTR (a population between 30,000 and 99,999)

^d^Large POPCTR (a population of 100,000 or more).

From the 1000 referrals sent to ConsultDerm, 9 different types of practitioners were identified to have sent the referrals (Table 3). The most common type of practitioner was family physicians (92.7%), followed by nurse practitioners (3.6%), pediatricians (1.5%), unknown specialties (1.2%), and internists (0.6%). The final 4 specialties, anesthesiology, emergency room physician, obstetrics and gynecology, and neurology, accounted for 0.4% of referrals.

**Table 3:** Distribution of Practitioner Type Among Those Sending Consults on www.consultderm.com.

Type of practitioner	N	Percentage (%)
Family physician	927	92.7
Nurse practitioner	36	3.6
Pediatrician	15	1.5
Unknown	12	1.2
Internist	6	0.6
Anesthesiologist	1	0.1
Emergency room physician	1	0.1
Obstetricians & Gynecologists	1	0.1
Neurologist	1	0.1

### Survey

**Figure 3:**
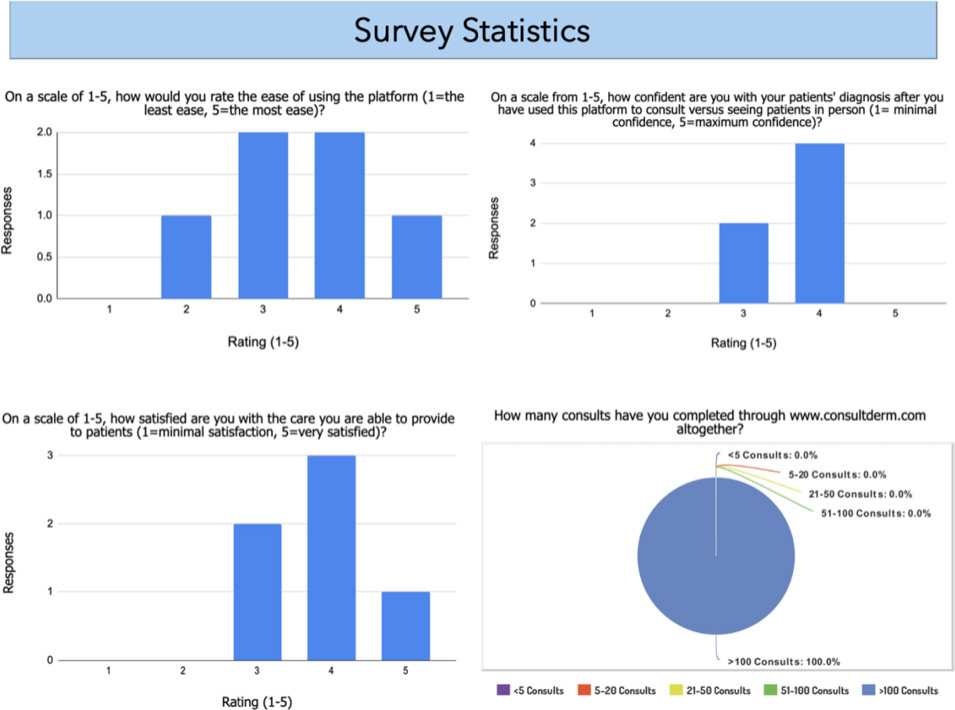
Summary of the survey statistics given to dermatologist respondents currently using the ConsultDerm platform to determine their experience in utilizing teledermatology. Please note that 6 out of the 7-dermatologist responded to the survey.

Regarding the survey, a total of 6 responses were collected out of 7 total requests (85.7% response rate) (Supplemental Table 1). All of the dermatologist respondents had completed more than 100 consults through the online teledermatology platform, ConsultDerm. Survey statistics, including ease of use of ConsultDerm, confidence with diagnosis through this platform, patient satisfaction, and the number of consults completed, can be found in Figure 3.

The survey also included an opportunity for the dermatologists to write in answers to questions. In terms of essential pieces of information necessary when receiving a photo to provide a diagnosis on, the respondents wrote the following: high-quality photographs that are in focus (close-up and far away), distribution of the rash, locations of lesion/rash, history, symptoms of the lesion (bleeding, itching), if the patient had any previous treatment, and personal/family history of similar rashes or lesions.

When asked if there were any specific health care settings where using a teledermatology platform would be most useful, 4/6 (66.7%) reported that implementation in remote or rural locations would be most beneficial. Respondents also reported that the barriers involved when trying to provide care through a teledermatology platform include: a lack of patient history, poor quality images and photos not representative of the problem, and time constraints in terms of taking a few days to respond.

Respondents were also asked how teledermatology could be improved. The answers included: easier way to transfer information from the teledermatology platform to an electronic medical record, training primary care to understand what can be achieved with the system, having an incentive system for the referring physician which may include a fee code to allow collection of good digital photos of the skin lesions as well as ensuring a detailed history is complete.

## Discussion

Our research study shows that teledermatology provides an unmet need for patients. Our data identified patient, clinical, and geographical information that may help guide future implementation of telemedicine services and further identify clinical education opportunities for referring practitioners.

In Canada, the average wait time to see a dermatologist for non-urgent medically insured visits is ten weeks.^11^ As previously mentioned, the average time for a response using ConsultDerm was only 5.2 days. This suggests that patient referrals for cutaneous diseases may be diagnosed more timely if telemedicine platforms were more regularly implemented into physician practice. The teledermatology platform could serve as either the primary method of consulting with a dermatologist or as a temporary measure until the patient receives an in-person appointment, depending on both patient preference and the confidence level of the dermatologist on their diagnosis and management plan, depending on the clinical situation.

When considering the general population and in-office visits, the types of consults seen through ConsultDerm were similar to what are commonly referred to for in-office visits. This may indicate that teledermatology and in-person office visits can be considered synonymous unless further in-person investigations are deemed necessary by the dermatologist. The majority of consults seen via ConsultDerm were as expected, with the most common diagnosis being dermatitis followed by acneiform conditions.

Notably, there were only 18 cases of skin cancer obtained through our analysis. This suggests that practitioners are referring patients for in-person appointments if a suspected cancerous lesion is seen or that the patients may have preferred an in-person visit. However, identifying the 18 cases of skin cancer in our pool of results, demonstrates the ability of teledermatology platforms to not only treat benign conditions in a timely fashion but also to identify skin cancers earlier rather than waiting for an in-office appointment, which could result in an emergency referral in comparison to a timelier wait.

Of the 1000 consults examined, we identified that almost half (45.4%) of those consults originated from small population centers and almost another half (41.2%) originated from large population centers, which indicates that more dense population areas also utilize teledermatology. This could be due to the long wait times required for in-person referrals or individual patients’ preferences based on their schedules.

To our surprise, we noticed that less than 1% of consults originated from rural areas. We anticipated that more consults would be seen from rural zones due to the lack of dermatologists located in the region. A rationale for this may be that most are either managed by family physicians or are seen in-person during in-city visits due to the smaller population requiring dermatology service. Additionally, the shortage of general practitioners available in rural areas may have inadvertently reduced the number of possible consults available from these regions. It would be valuable to investigate further into this, possibly surveying physicians of these regions regarding their awareness and comfort with using a teledermatology platform.

Table 3 identified varying types of practitioners who sent the referrals of our 1000 study subjects. Of those practitioners, a total of 92.7% (927) consisted of family physicians. This suggests an opportunity to provide more education to prospective and current family medicine physicians regarding what information is valuable when conducting a teledermatology referral and methods of taking photographs in a systematic manner such that they are useful to the consultant. In addition, our results indicate that specialists are not as active in using teledermatology, which may be due to the nature of their practice and its unnecessary need for dermatology referral. However, with dermatological concerns being a common contributor to ER visits, the lack of referrals from emergency physicians is surprising and perhaps indicates a lack of awareness of this platform. Assessing which specialists are commonly requesting dermatology referral and how teledermatology may be utilized within that specialty is an area that may warrant further investigation.

In Canada, there are several teledermatology platforms, including DermaGo, Dermcafe,Maple, and Consultderm. Some of these platform’s work based on practitioner-to-physician, requiring a patient to see a practitioner who will send background information and images to a dermatologist, as seen with ConsultDerm. While others, still requiring similar information to that seen in ConsultDerm, operate as a patient-to-physician platform, allowing patients to directly connect with dermatologists or family physicians with a dermatology interest. However, limitations to patient-to-physician platforms exist, especially when considering diseases that may be easily managed by general physicians or the possibility of neoplastic lesions, which may be misrepresented by a patient when reporting their symptoms.

The dermatologists who used the ConsultDerm platform had the freedom to use any teledermatology platform they preferred. Based on the survey responses from dermatologists using ConsultDerm, to have a successful teledermatology consult, we recommend that referring physicians provide a pertinent clinical history including duration of skin problem, symptoms, previous treatments, along with a personal/family history of dermatological conditions. We also emphasize that high-quality photographs from both close-up and far-away distances with various angles (head on, and side-to-side for example), would be of paramount importance. Further steps in improving teledermatology use in Canada may also include technological advances such as the centralization of electronic medical records, allowing the dissemination of patient information to be accessible by all relevant care providers. Additionally, creating a formalized training video for referring physicians would be valuable to ensure that the referring provider communicates all necessary information required by consultants. Although the dermatologists who participated in the survey reported possible ways to enhance the provider experience, the consensus by survey respondents was a positive experience using ConsultDerm. These survey respondents are regular users of the platform and it may be of value to have future respondents engage with other practitioner-to-physician dermatology platforms to have a more extensive report of user experience.

Although we identified several reasons why telemedicine in dermatology may provide benefit to both patients and practitioners, there exist several limitations in its administration. This includes incorrect diagnoses due to inaccurate and/or missing clinical information, poor quality images, and the lack of use of dermoscopy, which involves utilizing a handheld magnifying device (dermatoscope) to further analyze lesions. In addition, some referring physicians may not be comfortable with performing in-person biopsies, ultimately resulting in in-person referrals.

Limitations of our study include cases only being analyzed in Alberta. Data from more provinces would be warranted to have a more encompassing report of teledermatology. Furthermore, a systematic review including various teledermatology platforms could be performed in order to further elucidate the benefits of teledermatology and the various intricacies of each platform.

## Supplemental Material

Table S1 - Supplemental material for Implementation of Teledermatology in Alberta, Canada: A Report of One Thousand Cases Click here for additional data file. Supplemental material, Table S1, for Implementation of Teledermatology in Alberta, Canada: A Report of One Thousand Cases by Cristina Olteanu, Melika Motamedi, Jessica Hersthammer, Brandon Azer and Jaggi Rao in Journal of Cutaneous Medicine and Surgery

## References

[1] Campagna M. Naka F. Lu J . Teledermatology: an updated overview of clinical applications and reimbursement policies. Int J Womens Dermatol. 2017;3(3):176-179. 10.1016/j.ijwd.2017.04.002 28831431 PMC5555283

[2] Lee KJ. Finnane A. Soyer HP . Recent trends in teledermatology and teledermoscopy. Dermatol Pract Concept. 2018;8(3):214-223. 10.5826/dpc.0803a013 30116667 PMC6092076

[3] Chawla S . COVID-19: challenges and opportunities for dermatology response. J Dermatolog Treat. 2020;31(4):326. 10.1080/09546634.2020.1751040 32241193

[4] Finnane A. Dallest K. Janda M. Soyer HP . Teledermatology for the diagnosis and management of skin cancer: a systematic review. JAMA Dermatol. 2017;153(3):319-327. 10.1001/jamadermatol.2016.4361 27926766

[5] Canadian Medical Association . Dermatology Profile [Internet]. https://www.cma.ca/. [cited 2022 Feb 11]. https://www.cma.ca/sites/default/files/2019-01/dermatology-e-v2.pdf

[6] Coustasse A. Sarkar R. Abodunde B. Metzger BJ. Slater CM . Use of teledermatology to improve dermatological access in rural areas. Telemed J E Health. 2019;25(11):1022-1032. 10.1089/tmj.2018.0130 30741608

[7] Lee JJ. English JC . Teledermatology: a review and update. Am J Clin Dermatol. 2018;19(2):253-260. 10.1007/s40257-017-0317-6 28871562

[8] Datta SK. Warshaw EM. Edison KE et al. Cost and utility analysis of a store-and-forward teledermatology referral system: a randomized clinical trial. JAMA Dermatol. 2015;151(12):1323-1329. 10.1001/jamadermatol.2015.2362 26375589

[9] Zakaria A. Maurer T. Su G. Amerson E . Impact of teledermatology on the accessibility and efficiency of dermatology care in an urban safety-net hospital: a pre-post analysis. J Am Acad Dermatol. 2019;81(6):1446-1452. 10.1016/j.jaad.2019.08.016 31415834

[10] Dictionary, Census of Population, 2016 - Population centre (POPCTR) . Government of Canada, Statistics Canada. 2016. https://www12.statcan.gc.ca/census-recensement/2016/ref/dict/geo049a-eng.cfm

[11] Chow EY. Searles GE . The amazing vanishing Canadian dermatologist: results from the 2006 Canadian dermatology association member survey. J Cutan Med Surg. 2010;14(2):71-79. 10.2310/7750.2010.09025 20338122

