# Supplementary material for: Implementation of Teledermatology in Alberta, Canada: A Report of One Thousand Cases
Source: J Cutan Med Surg. 2022 Jul 8;26(5):477–84. doi: 10.1177/12034754221108990 (PMC9476229; doi:10.1177/12034754221108990)
Supplement: Table S1 - Supplemental material for Implementation of Teledermatology in Alberta, Canada: A Report of One Thousand Cases [file sj-pdf-1-cms-10.1177_12034754221108990.pdf]

Table S1: Survey Monkey questions and possible answers. A total of 9 quantitative and qualitative questions were distributed to 7 dermatologists via the platform Survey Monkey.

Question	Possible Answers	Responses
1. How many consults have you completed through www.consultderm.com altogether?	<5<br>5-20<br>20-50<br>50-100<br>>100	>100
2. On a scale of 1-5, how would you rate the ease of using the platform?	1=the least ease<br>5=the most ease	3.5
3. On a scale of 1-5, how satisfied are you with the care you are able to provide to patients?	1=minimal satisfaction<br>5=very satisfied	3.8
4. On a scale from 1-5, how confident are you with your patients' diagnosis after you have used this platform to consult versus seeing patients in person?	1=minimal confidence<br>5=maximum confidence	3.7
5. Are there any specific health care settings for patients where you find that using this platform is the most useful?	Community<br>Hospital<br>Long-Term Care	• Remote or rural locations<br>• Patients who live in areas without access to dermatology locally
6. What are the three top conditions you treat through the platform?	Open-Ended Response	• Dermatitis<br>• Atypical Nervi<br>• Skin Cancer<br>• Psoriasis<br>• Alopecia<br>• Seborrheic keratoses<br>• Drug reactions<br>• Contact allergies<br>• Birth marks
7. What are the top three pieces of information you would deem necessary when receiving a photo to provide a diagnosis on?	Open-Ended Response	• High quality photos<br>• Location/Distribution<br>• History<br>• Previous treatments<br>• Symptoms
8. What are three barriers you find when trying to provide care through a platform such as this?	Open-Ended Response	• Poor quality photos<br>• Lack of adequate history or accurate patient identifiers<br>• Not being able to palpate the rash or to complete a physical examination<br>• Time constraints
9. How could teledermatology be improved? (eg, Patient follow-up, System accessibility)	Open-Ended Response	• Easier way to transfer information from the teledermatology platform to an electronic medical record.<br>• Training primary care to understand what can be achieved with the system,

		• Having an incentive system for the referral physician such as a fee code to take more time for a comprehensive history<br>• Instructions for good digital photos of the skin lesions<br>• Improved accuracy of patient demographics and history

Table S2: Specific diseases encountered on ConsultDerm. The various diseases were categorized into general conditions as indicated in Table 2.

**Conditions** (as indicated in Table 2)	**Specific disease**
Dermatitis	• Dermatitis<br>• Contact dermatitis<br>• Impetiginized dermatitis<br>• Nummular dermatitis<br>• Dyshidrotic dermatitis<br>• Atopic dermatitis<br>• Asteatosis dermatitis<br>• Lichen simplex chronicus<br>• Atopic eruption of pregnancy<br>• Pityriasis alba<br>• Prurigo nodularis
Acneiform conditions	• Acne vulgaris<br>• Acne keloidalis nuche<br>• Nodulocystic acne<br>• Acne conglobata<br>• Folliculitis<br>• Pseudofolliculitis<br>• Lichen spinulosus<br>• Rosacea<br>• Hidradenitis suppurativa<br>• Keratosis pilaris<br>• Open comedone, benign
Benign lesions/neoplasms	• Seborrheic keratosis<br>• Nevus<br>• Cysts<br>• Pyogenic granuloma<br>• Lichenoid keratosis<br>• Angiokeratomas<br>• Portwine stain<br>• Angiomas<br>• Actinic keratosis<br>• Irritated keratosis<br>• Callous<br>• Dermatosis papulosa nigra<br>• Benign adnexal tumor<br>• Ectopic sebaceous glands<br>• Focal skin atrophy<br>• Keratoacanthoma

	• Eccrine poroma<br>• Sebaceous adenoma<br>• Sebaceous gland hyperplasia<br>• Acrochordons<br>• Porokeratoses<br>• Lentigo/solar lentigo<br>• Congenital pigmented lesion
Infections	Fungal<br>• Tinea capitis<br>• Tinea corporis<br>• Tinea pedis<br>• Tinea versicolor<br>• Tinea incognito<br>• Tinea cruris<br>• Thrush<br>• Kerion<br>• Onychomycosis<br>Viral<br>• Warts (ex. verruca plantaris, verruca vulgaris etc).<br>• Cutaneous horn<br>• Herpes simplex virus (HSV) infection<br>• Shingles<br>• Molluscum contagiosum<br>• Erythema multiforme<br>• Herpetic whitlow<br>• Viral exanthem<br>Bacterial<br>• Furunculosis<br>• Impetigo<br>Ectoparasites<br>• Scabies
Dyspigmentation	• Hyperpigmentation<br>• Vitiligo<br>• Melasma<br>• Acanthosis nigricans<br>• Benign/idiopathi guttate hypomelanosis<br>• Poikiloderma
Vasculopathy/ vasculitis/ purpura	• Vasculopathy<br>• Vasculitis<br>• Benign purpura<br>• Cutaneous vascular dilatation, benign<br>• Ecchymosis, possibly due to underlying vascular lesion<br>• Hemosiderin pigmentation<br>• Venous stasis changes<br>• Livedo reticularis<br>• Vascular dilatation/lesion
Other Papulosquamous	• Seborrhoeic dermatitis<br>• Lichenoid dermatitis<br>• Pityriasis rosea<br>• Lichen planus<br>• Lichen striatus
Psoriasis	• Psoriasis

	• Palmoplantar psoriasis<br>• Guttate psoriasis<br>• inverse psoriasis<br>• Plaque psoriasis
Other inflammatory skin diseases	• Lichen sclerosus<br>• Miliaria<br>• Balanitis<br>• Cheilosis<br>• Discoid lupus erythematosus<br>• Perniosis/chilblainlike reaction<br>• Polymorphous light eruption
Skin cancer	• Basal cell carcinoma<br>• Squamous cell carcinoma
Nail disease (non-infectious)	• Dystrophy<br>• Nail dermatitis<br>• Koilonychia<br>• Leukonychia<br>• Geometric nail pitting secondary to alopecia areata<br>• Beau's lines, benign<br>• Nail trauma with onychomycosis<br>• Melanonychia striata
Urticaria	• Urticaria<br>• Solar urticaria<br>• Upper lip swelling
Alopecia	• Alopecia areata<br>• Androgenic alopecia<br>• Scaring alopecia<br>• Female pattern hair loss
Granulomatous	• Granulomatous dermatitis<br>• Granuloma annulare
External Trauma and Scars	• Hemorrhagic scar<br>• subungual hemorrhage<br>• Subcorneal hemorrhage
Bullous disease	• Bullous pemphigoid
Fibromatoses	• Dermatofibroma<br>• Early Dupuytren's contracture<br>• Knuckle pads<br>• Palmoplantar keratoses
Oral conditions	• Black hairy tongue
Hypersensitivity reaction	• Erythema multiforme<br>• Arthropod bite reaction/Papular urticaria<br>• Erythema nodosum
Other	• Plantar xerosis<br>• Macular amyloidosis<br>• Uremic pruritus with excoriations<br>• Porphyria cutanea tarda<br>• Full thickness ulceration<br>• Nonspecific ulceration/ulceration
